# Medical Students' Perceptions of Patient-Doctor Relationship in South Korea: Concept Mapping Analysis

**DOI:** 10.3389/fpubh.2021.658220

**Published:** 2021-11-16

**Authors:** So Jung Yune, Seung Hee Kang, Kwihwa Park

**Affiliations:** ^1^Department of Medical Education, School of Medicine, Pusan National University, Busan, South Korea; ^2^Department of Lifelong Education and Counseling, Pukyong National University, Busan, South Korea; ^3^Department of Medical Education, Gachon University College of Medicine, Incheon, South Korea

**Keywords:** medical student, patient-doctor relationship, concept mapping, medical education, patient-centered care

## Abstract

**Introduction:** The patient-doctor relationship has evolved from early paternalism to a consumerism and partnership model that emphasizes cooperation. Patient-doctor relationships might vary with the socio-cultural environment, because the medical environment affects such relationships.

**Method:** We investigated the patient-doctor relationship among medical students through concept mapping analysis. Twenty-six fourth-grade Korean medical students wrote a reflection journal and participated in the concept classification and the importance evaluation of the derived concept. ALSCAL multidimensional scaling and Ward hierarchical cluster analysis were performed. Also, the 5-point Likert scale was used to evaluate the importance of the concept.

**Results:** Sixty-six statements about the patient-doctor relationship were extracted and grouped into six clusters. The x-axis is the dimension of “Information-Respect,” and the y-axis is “Changeability-Persistence.” Six patient-doctor concepts were derived and students evaluated “Patient-centered” as the most important.

**Conclusions:** Medical students express various concepts of the patient-doctor relationship. Considering that they may encounter various medical conditions and patients, it is necessary that they understand deeply the complex patient-doctor relationship.

## Introduction

The relationship between doctors and patients is one of the most difficult among interpersonal relationships. This relationship is sometimes involuntary and emotionally laden, and at the same time deals with life-related issues, so close cooperation is required (1). The quality of doctor–patient interaction and communication is the most important factor in the quality of medical care and plays a fundamental role in the medical care process (2).

Patient-doctor relationships have evolved from early paternalism to a consumerism and partnership model that emphasizes cooperative relationships (3). Traditionally, doctor–patient relationships have followed a paternalistic and vertical mode in which doctors try to achieve the best outcomes for patients, and a good patient is one who listens to his/her doctor faithfully. To put it simply, the doctor ordered, and the patient obeyed (4). As medical care has changed to a service perspective, a service or consumer model has appeared in which doctors are the service providers and patients are perceived as consumers. Perceptions of the relationship between patient and doctor have shifted toward a view of the relationship as contractual. In addition, respect for individual autonomy and patients' right to self-determination have become important factors, and patients became active consumers who actively pursue their rights (5). The partnership model regards collaborative efforts between the two equal partners as important. It is assumed that in order to achieve successful treatment results, both work together and complement each other. Patients are respected as mature people who make their own decisions on the basis of the principle of autonomy (3). Recently, in patient-doctor relationships, the concepts of participatory decision-making, shared decision-making and patient centrality have been becoming increasingly important (5, 6). With the development of medical technology and information and communication technology, it is easy to acquire health and medical information, and the patient-doctor relationship is developing into an equal and interactive holistic relationship (4).

The relationship between the doctor and the patient is the product of the two participants' attitudes toward each other based on communication (7), so the doctor's behavior may vary depending on how the doctor thinks about his/her patient (8). Because people's behavior is influenced by their orientation to others and by their perceptions (9). In Korea, there is a difference in mutual perception that doctors want their patients to be treated as professionals regardless of their age, and their patients who are older than them want their doctors to treat them as elders (10). The doctor-patient relationship is understood as a matter of power and conflicts are sometimes induced (11). Therefore, in order to build a good patient-doctor relationship, how the doctor perceives the relationship with the patient is an important factor; therefore, scholars need to pay attention to this issue.

Medical schools have emphasized communication skills, professionalism, and humanities and sociology education in order to establish the requisite patient-doctor relationships. However, few studies to date have examined medical students' perception of the patient-doctor relationship. Especially in Korea, the study of the patient-doctor relationship has focused on communication skills (12, 13) and trust (M15), which are factors that influence relationships. These studies have limitations in identifying the essential characteristics of patient-doctor relationships, so it is necessary to understand the characteristics of the patient-doctor relationship itself. Eveleigh et al.'s study (14) also pointed out that the relationship between doctors and patients has not yet been fully conceptualized. In particular, understanding medical students' perceptions of the patient-doctor relationship is especially important in that they can predict future changes in the health care environment.

Patient-doctor relationships might vary with the socio-cultural environment, because the medical environment affects such relationships (15, 16). In the United States, a cooperative patient-doctor relationship based on individualism, autonomy, and value of service prevails. However, in many Asian countries, the patient-doctor relationship is hierarchical (17), the doctor is likely to be a virtuous authoritarian figure who is caring and responsible for the welfare of patients. In return, he/she obtains a high level of regard (3). Especially in Korea, there are few people with their family doctor, so the concept of the patient-doctor relationship may be different from that of Western countries, and many medical students have a doctor-centered attitude, according to some studies (18, 19). However, most of the studies have sought to investigate the patient-centered attitude of doctors using PPOS (Patients Practicer Orientation Scale) (7) developed in the United States (19–21), Therefore, there is a limit to identifying how medical students perceive the patient-doctor relationship in Korea.

One of the research methods for understanding the perception of the patient-doctor relationship is concept mapping, which is a particularly suitable method for mapping complex, not yet fully crystallized topics, into underlying concepts (22). This method is a participatory methodology used to visually represent the ideas or thoughts of an individual or group, and it is suitable for analyzing responses to open-ended survey questions (23). The participants in the concept-mapping study create ideas that constitute the content of the concept mapping, provide information on the relationship between each idea, and participate in interpreting the results of the generated concept mapping. In this expert-based concept-mapping method, the quantitative analysis of multidimensional scaling and hierarchical cluster technique and qualitative analysis of knowledge structure are combined to provide a structural identification of the mental model of experts (24). Therefore, concept mapping is an effective method to analyze how medical students perceive the patient-doctor relationship.

The aim of the present study is to investigate the concept of the patient-doctor relationship and to examine the overall characteristics, status, and relations among concepts within the patient-doctor relationship by using concept mapping. This will help us to understand how Korean medical students conceive the patient-doctor relationship now. Furthermore, it will be a basic study for the development of the requisite educational programs about patient-doctor relationships and of a questionnaire to survey them.

The specific research questions are as follows. First, how do medical students perceive the doctor–patient relationship? Second, what is the relative importance of the concepts related to the doctor–patient relationship as perceived by medical students?

## Methods

### Research Design

This study was conducted in accordance with Kane and Trochim's (25) suggested six-step study process for a concept map: (1) preparation, (2) generation of statements, (3) structuring of statements, (4) representation of statements, (5) interpretation of concept maps, and (6) integration of maps. In the first stage, a focus question was drafted in preparation for conceptual study, and research participants were selected for data collection. The second stage was the idea-generation stage. All participants were asked to write one reflection essay based on their experience of the patient-doctor relationship, and ideas were generated by writing five or more definition statements for the patient-doctor relationship. In the third stage, in order to structure the statements, 66 cards with statements were made so that the participants in the study were grouped into the same group as “the statements that seem to be together.” However, only one statement could not be classified into one group, and all the given statements could not be classified into one group (25). Students conducted their own classification work only once in a place designated by the researcher. In step 4, the results classified by the study participants were coded as zero and one to create a group similarity matrix (GSM) (26). In addition, the dimension was determined by multidimensional scaling (MDS), and a hierarchical cluster analysis was performed with the calculated x- and y-values. A hierarchical cluster analysis was performed to divide the statements on the map into an internally consistent group to draw a conceptual map. Ward's hierarchical clustering makes distance-based data more meaningful (25). In step 5, the conceptual diagram was interpreted using statements, cluster lists, and cluster maps. The dimension name of the cluster map was set, and the concept of the cluster was interpreted. To determine the relative importance of the final derived concept cluster, an online survey was conducted of research participants, and an analysis was conducted of technical statistics. Finally, the medical education implications of the patient's relationship were derived from the concept map.

### Participants

We announced the recruitment of research participants to fourth-grade students who had experienced clinical practice for more than one year. We explained the research methods and procedures to 26 students who were interested in the research. Students who agreed to participate in the study were required to complete a consent form. Among the 26 students who participated in the study, 14 (53.8%) were males; 12 (46.2%) were females; and 21 (80.8%) were age 25 and older. Moreover, 22 (84.6%) students had attended medical school after going through premedical school. Table 1 provides demographic information on our participants across the concept-mapping process.

**Table 1:** Participant demographics.

**Demographics**		***N* (%)**
Gender	Male	14 (53.8%)
	Female	12 (46.2%)
Age	25~26	21 (80.8%)
	27~	5 (19.2%)
Experience	Premedical school experience	22 (84.6%)
	No premedical school experience	4 (15.4%)

### Instruments

The research participants were asked to “write freely about the patient-doctor relationship, what felt or learned in the case of a patient experienced directly or indirectly during the past year's clinical clerkship,” and the reflective essay was required to be more than two pages in length. In addition, I tried to provide more than five understandings of the patient-doctor relationship. All data were received directly by the researcher via email. Furthermore, an online questionnaire was conducted to evaluate the relative importance of the concept cluster and the statements included in the cluster as the final stage of the study on a 5-point Likert scale.

### Analysis

Among the statements derived from the reflective essays and the definition of patient-doctor relationship written by medical students, the process of selecting only the responses of two or more people, synthesizing and editing the duplicates was done by the researchers, and the final 66 statements were made. In addition to the researchers, two educational experts (Education PhD., more than 10 years of teaching experience in medical education) participated in the statement-classification work to confirm whether the statement-generation process was reasonable. And each statement was made in the form of a card so that the research participants could classify their ideas. Each of the 26 study participants who were medical students individually classified 66 statements into groups according to similarity. The number of groups ranged between 11 and 29. A GSM was created from 66 statements derived from the collected data, and ALSCAL MDS was used to place each statement as a separate point on the conceptual map. In addition, Ward- hierarchical cluster analysis (HCA) was performed to divide points on the map into internally consistent clusters. The SPSS 25.0 program was used for GSM, MDS, HCA, and descriptive statistical analysis.

## Results

### Patient-Doctor Relationship Concept

Concept mapping analysis was performed to investigate the perception of medical students on the patient-doctor relationship. Twenty-six study participants grouped the 66 finalized patient-physician relationship statements. MDS results showed that the agreement indices in two and three dimensions were 0.35 and 0.26, respectively, to meet the agreement index range of Kane and Trochim (25). 'Kane and Trochim (25) suggested to represent in 2D for the efficiency of conceptual diagram interpretation, and suggested the appropriate level of agreement as 0.205 to 0.365' in Manuscript.

**Figure 1:**
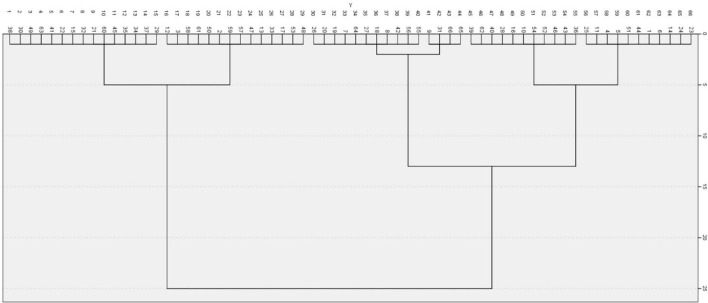
Dendrogram by cluster analysis (Ward).

We decided in two dimensions considering the interpretability and efficiency. Table 2 shows the stress value and R^2^ for each dimension of the GSM by the multidimensional scale analysis. A ward hierarchical cluster analysis was conducted to determine how 66 statements were classified in two-dimensional space. As a result of a dendrogram (Figure 1), the number of clusters could be classified into two, three, or six clusters. Finally, six clusters were decided upon, because there was a clear difference in meaning among clusters.

**Table 2:** Stress value and R^2^ by dimension.

**Dimensions**	**Stress value**	**R^**2**^**	**ΔR^**2**^**
1	0.59	0.23	-
2	0.35	0.44	0.21
3	0.26	0.59	0.15
4	0.20	0.67	0.08
5	0.16	0.75	0.08
6	0.13	0.81	0.06

**Figure 2:**
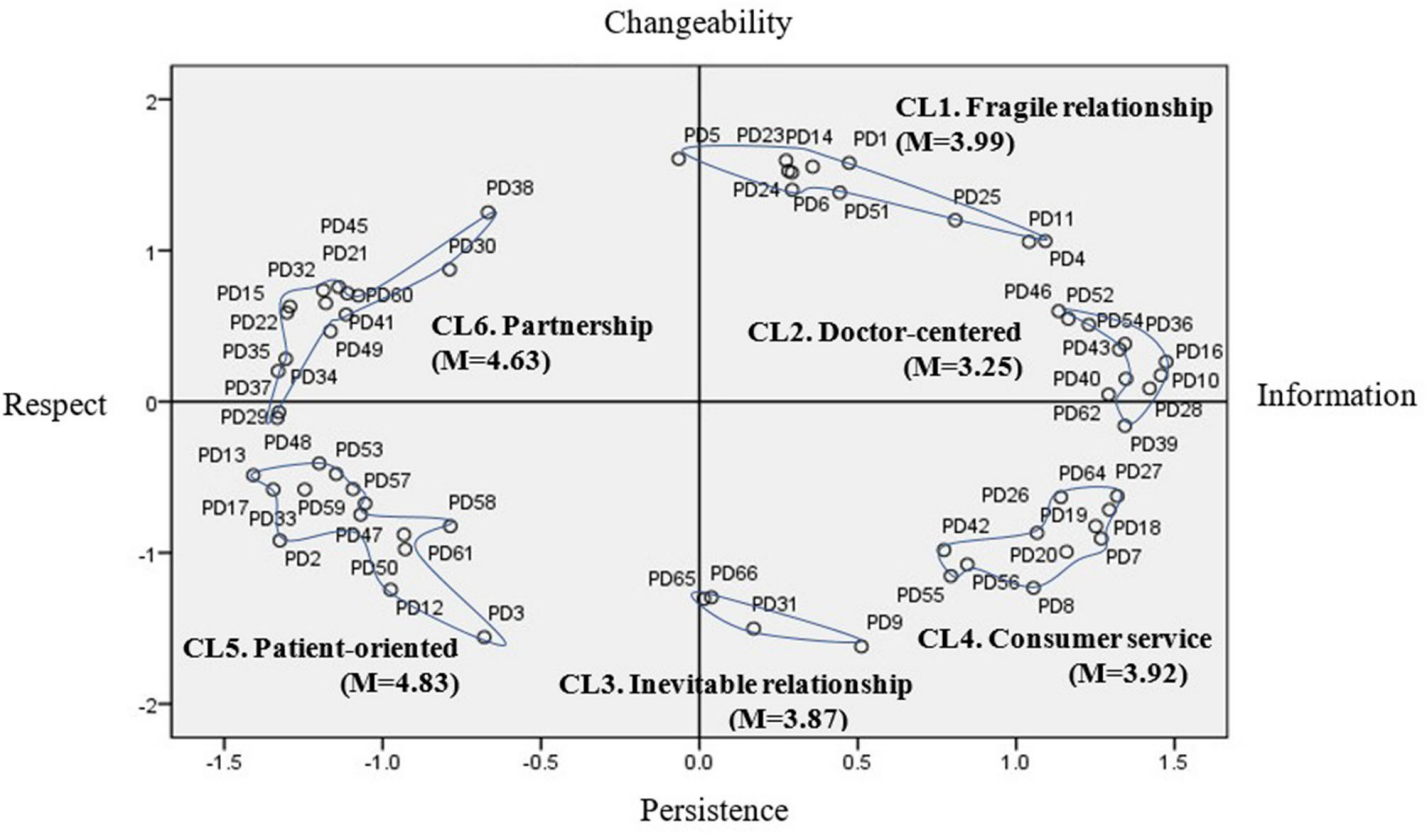
Medical students' concept map of patient-doctor relationship.

Based on the location and content of the statement, the x-axis is the “Information-Respect” dimension, and the y-axis is named the “Changeability-Persistence” dimension as shown in Table 3. After each cluster's statements were reviewed, each cluster was named to reflect its characteristics in Figure 2. Cluster 1 contains 11 statements and refers to a relationship that can be changed depending on the patient, doctor, or therapeutic situation. Therefore, Cluster 1 was called “Fragile relationship.” Cluster 2 was named “Doctor-oriented” and contains 11 statements. Cluster 2 is a traditional concept of patient communication, meaning a relationship led by a doctor with information. Cluster 3 contains four statements and refers to the “Inevitable relationship”. Cluster 3 emphasizes that anyone can be a patient, and the relationship with the doctor is inevitable. Cluster four means “Consumer service”. It consists of 11 statements and refers to a relationship separated into distinct roles within the medical system, such as providing services to consumers. Cluster 5 is “Patient-centered”, which refers to a patient-centered therapeutic relationship based on empathy and trust and consists of 14 statements. Cluster 6, “Partnership”, consists of 15 statements, the largest number. Cluster 6 means a relationship that cooperates with interdependence in a treatment situation as a collaborative relationship. The statements classified into six clusters are as shown in Table 4.

**Table 3:** Statement located at the extreme end of the X-Y axis.

**(+) Information**	**(-) Respect**
**X-axis: 'Information-Respect'**	
16. The patient-doctor relationship is the same as teacher and student relationship. 10. The patient-doctor relationship is a one-way relationship.	13. In patient-doctor relationships, caring for each other is fundamental. 17. The patient-doctor relationship requires empathy.
**(+) Changeability**	**(-) Persistence**
**Y-axis: 'Changeability-Persist'**	
5. Medical services may vary depending on the level of information the patient has. 23. The patient-doctor relationship is constantly changing.	9. The patient-doctor relationship is a one-to-many relationship. 3. There must be no lies to maintain the patient-doctor relationship.

**Table 4:** Clusters and statements.

**No**.	**Statement**	***M (SD)***
**CL1. Fragile relationship**		**3.99 (0.503)**
4	Providing the right treatment direction is important to maintain patient-doctor relationship.	4.33 (0.482)
14	The patient-doctor relationship is an important factor influencing treatment.	4.29 (0.955)
44	Patient-doctor relationships vary depending on the patient or doctor.	4.08 (0.654)
51	The patient-doctor relationship is one of the most important factor in determining the outcome of treatment.	3.88 (0.992)
6	The patient-doctor relationship is a relationship that changes depending on the social situation.	3.88 (0.741)
1	The patient-doctor relationship plays an essential role in establishing the treatment direction.	3.71 (1.042)
23	The patient-doctor relationship is constantly changing.	3.67 (0.816)
11	The patient-doctor relationship is selectable.	3.58 (0.83)
24	The patient-doctor relationship is a fragile relationship.	3.33 (0.917)
5	Medical services may vary depending on the level of information the patient has.	3.29 (1.16)
25	The patient-doctor relationship is like a tug of war.	2.63 (0.875)
**CL2. Doctor-oriented**		**3.25 (0.442)**
40	The patient-doctor relationship is like a guide and traveler.	4.33 (0.482)
46	The patient-doctor relationship is like a caregiver and a child.	4.29 (0.955)
10	The patient-doctor relationship is a one-way relationship.	4.08 (0.654)
16	The patient-doctor relationship is the same as teacher and student relationship.	3.88 (0.992)
52	The doctor is responsible for the patient's health.	3.88 (0.741)
54	This is an unbalanced relationship because doctors have more information and skills than patients do.	3.71 (1.042)
36	A patient-doctor relationship can be vertical when the doctor is reliable.	3.67 (0.816)
62	The balance of strength is uneven in the patient's medical relationship.	3.58 (0.830)
43	The patient-doctor relationship is vertical.	3.33 (0.917)
28	The patient-doctor relationship is like a sponsorship relationship.	3.29 (1.160)
39	The patient-doctor relationship is like an information provider and a decision maker.	2.63 (0.875)
**CL 3. Inevitable relationship**		**3.87 (0.797)**
31	Everyone has no choice but to see a doctor at least once.	4.13 (0.900)
66	The patient-doctor relationship is persistent.	4.00 (0.722)
9	The patient-doctor relationship is a one-to-many relationship.	3.67 (1.167)
65	Patients and doctors have a long-term relationship.	3.63 (0.875)
**CL4. Consumer service**		**3.92 (1.018)**
8	The patient-doctor relationship is closely related to the medical system.	4.13 (0.850)
55	For a doctor, a patient can be one of several, but for a patient, a doctor can be everything.	3.92 (0.974)
18	The patient-doctor relationship is a relationship that requests and solves problems.	3.79 (0.779)
7	The patient-doctor relationship is the process of delivering medical services.	3.79 (0.658)
27	The patient-doctor relationship is the same as a health care provider and a beneficiary.	3.67 (0.868)
64	The patient-doctor relationship is a producer-consumer relationship.	3.58 (0.776)
26	The patient-doctor relationship is a counselor-client relationship.	3.58 (0.654)
56	The patient-doctor relationship is a society-mediated relationship.	3.50 (0.834)
42	Patient expresses and the doctor observes.	3.50 (0.933)
19	The patient-doctor relationship is similar to the consumer-consultant relationship.	3.29 (0.859)
20	The patient-doctor relationship is a business.	3.21 (1.021)
**CL5. Patient-oriented**		**4.83 (0.482)**
61	Patients and doctors must trust each other.	4.50 (0.590)
12	In patient-doctor relationships, trust is fundamental.	4.42 (0.584)
53	Patients and doctors need to understand each other.	4.33 (0.482)
17	The patient-doctor relationship requires empathy.	4.29 (0.690)
2	Patients and doctors need to think from each other's point of view	4.17 (0.637)
50	Patients and doctors must be honest and truthful with each other.	4.17 (0.761)
58	Patients and doctors are obliged to obey each other.	4.17 (0.761)
48	The patient-doctor relationship must be able to lean against each other.	3.92 (0.654)
3	There must be no lies to maintain the relationship between the patient and the doctor.	3.92 (0.830)
13	In patient-doctor relationships, caring for each other is fundamental.	3.79 (0.833)
47	In a patient doctor's relationship, you should always think from each other's perspective.	3.71 (0.908)
57	Emotional consensus is needed in patient-doctor relationships.	3.71 (0.624)
59	In patient-doctor relationships, they interact with each other.	3.71 (0.751)
3	The patient-doctor relationship is sometimes friendships that can soothe each other's loneliness.	2.58 (1.018)
**CL6. Partnership**		**4.63 (0.495)**
37	Patients and doctors must respect each other.	4.46 (0.509)
29	Two-way communication is needed in patient-doctor relationships.	4.38 (0.576)
15	The patient-doctor relationship is cooperative.	4.38 (0.647)
49	Both the doctor and the patient should work for a good relationship.	4.33 (0.761)
30	Patients and doctors are partners for problem solving.	4.17 (0.482)
22	Patients and doctors are relationships that give each other feedback.	4.00 (0.722)
60	The patient-doctor relationship is mutually beneficial.	4.00 (0.722)
41	The patient-doctor relationship is complementary.	3.92 (0.830)
63	The patient-doctor relationship is interdependent.	3.87 (0.680)
21	The patient and the doctor are companions to the same purpose.	3.75 (0.608)
35	The patient-doctor relationship is supportive of each other.	3.62 (0.875)
32	Patients and doctors learn from each other and grow.	3.58 (0.929)
45	The patient and the doctor are in a horizontal relationship.	3.33 (0.963)
34	The relationship between the patient and the doctor is the same as the normal person-to-person relationship.	3.29 (1.083)
38	The patient-doctor relationship is peaceful.	2.79 (1.215)

### Ranking of Clusters and Statements

The importance of each statement was evaluated according to the 5-point Likert scale in Table 2. These results indicate the relative importance of each cluster. Medical students who participated in the study rated “Patient-centered” (*M* = 4.83, *SD* = 0.482) as the most important. Next came “Partnership” (*M* = 4.63, *SD* = 0.495), followed by “Fragile relationship” (*M* = 3.99, *SD* = 0.503), “Consumer service” (*M* = 3.92, *SD* = 1.018), “Inevitable relationship” (*M* = 3.87, *SD* = 0.797), and “Doctor-oriented” (*M* = 3.25, *SD* = 0.442).

## Discussion

The modern patient-doctor relationship has changed for various reasons. As patients have had easy access to medical information through various media including the internet, they have taken more of a leadership position in the diagnosis and treatment of diseases than in the past. In recent years, the concept of patient-centered care has received increased attention worldwide (2). However, the doctor–patient relationship is influenced by the socio-cultural context (3, 27). This study aims to analyze the concept of the doctor–patient relationship among medical students in Korea.

The patient-doctor relationship derived from this study was divided into two dimensions: “Information-Respect” and “Changeability-Persistence.” As a result of multidimensional scaling, the concept of the patient-doctor relationship recognized by medical students was found to consist of six clusters. Cluster 1 was called “Fragile relationship.” Cluster 2 was named “Doctor-oriented.” Cluster 3 refers to the “Inevitable relationship.” Cluster 4 means “Consumer service.” Cluster 5 is “Patient-centered.” Cluster 6 is “Partnership.” Each concept consisted of separate statements for each cluster.

Cluster 1, “Fragile relationship,” containing 11 statements, refers to a relationship that can be changed depending on the patient, doctor, or therapeutic situation. In Korea, it is not as if the patients are constantly being taken care of by their doctor when they get sick or visit a hospital for treatment. Furthermore, if they want to hear other doctors' opinions about the treatment they have received, they can easily “doctor shop” at their choice (15). Therefore, medical students can recognize that their relationship with patients is changeable and fragile. These results are also shown in the statement in cluster 1; for example, providing the right treatment direction is important to maintain the patient-doctor relationship, and patient-doctor relationships vary depending on the patient or doctor. The relationship between the patient and the doctor is constantly changing. This is a concept that can be considered in the Korean medical context. It reflects the somewhat personal aspect of modern medicine (3).

Cluster 2 is a doctor-centered relationship, the same concept as the traditional paternalism of a patient-doctor relationship. Paternalism was defined as a more biomedical, clinician-centered approach in which the patient was less involved and more passive and the provider controlled the interaction (28). From a paternalistic point of view, patient-doctor relationships are considered parent–child relationships in which parents are relied upon to make decisions. The statement also includes the relationship of guide and traveler, caregiver and child, and teacher and student. This means that the doctor controls the situation. This relationship is often considered authoritative and directive because of the imbalance of knowledge. This may be thought of as an undesirable perception of the past, but it is also claimed that paternalism is needed for treatment and health recovery (29). Cluster 3 contains four statements and refers to the “Inevitable relationship.” It emphasizes that anyone can be a patient, and the relationship with the doctor is inevitable. This is suitable for the situation in which the number of patients with chronic diseases who need prevention and continuous care is increasing at a rapid rate (7). It is in line with the fact that for patients with chronic disease the relationship between the patient and the doctor has lasted longer than in the past (30).

Cluster four means “consumer” from a consumer-service perspective (31). It consists of 11 statements and refers to a relationship separated into distinct roles within the medical system, such as providing services to consumers. The patient-doctor relationship is closely related to the medical system. The patient-doctor relationship is about the process of delivering medical services. It can be seen that medical students recognize the relationship with patients from the service point of view. This means that asymmetric information of information has been improved, and patients have shared medical information and started to change from being passive consumers to being active consumers (32). In Korea, the concept of consumer sovereignty has long been expanding, and in December 2004, the bill of patient rights was enacted; patients began to be regarded as medical service consumers rather than as non-power groups, and doctors as medical service providers rather than as power groups. As a result, patients have only sought care from doctors to treat illness in the past, but today patients are expecting a holistic and equal medical–patient relationship and better medical services beyond treatment (33).

Cluster 5 is “patient-centered”, and cluster 6 is “partnership”. The two concepts can be interpreted in the same context through the principle that “patient-centered care is a model for collaborative medical interactions” (34). The term “patient centeredness” should be reserved to describe a moral philosophy with three core values: first, to consider patients' needs, wants, perspectives, and personal experiences; second, to provide information related to patients' treatment and to provide patients with the opportunity to participate in their care; third, to improve partnership and understanding in the patient-doctor relationship (6, 31). Cluster 5 emphasizes the first of these values, and cluster 6 emphasizes the second and third values.

In other words, statements included in the patient-centered relationship of cluster 5 mean that the patient-centered relationship requires trust. For example, patients and doctors must trust each other; patients and doctors need to understand each other; and the patient-doctor relationship requires empathy. Thus, medical students refer to respect, trust, and consideration for patient needs. Some statements in the partnership relationship of cluster 6 emphasize the need for the patient and doctor to respect each other. Two-way communication is needed in patient-doctor relationships. The patient-doctor relationship is cooperative. As such, it includes more patient-centered care and emphasizes the partnership and cooperation of doctors and patients. In addition, the patient-centered relationship in this study is located in the Respect-Changeable Quadrant, which emphasizes an immediate and changeable response to the patient's needs. The partnership relationship of cluster 6 is at the level of Respect-Persistence. There is little difference in that it emphasizes a more continuous cooperative relationship in the treatment situation.

As above, despite changes in the concept of the patient-doctor relationship according to the situation of the times, the subjects of this study recognized various aspects of the patient-doctor relationship. This supports the argument that medical education should teach students how to respond carefully to patients' values, interests, and diverse styles and to align their behavior to patients' orientation rather than to teach students a specific approach, behavior, or skill involved in patient-doctor relationships (17). In addition, it is similar to the argument that the importance of a patient-centered relationship and patient-centered communication has been emphasized recently, but patient-centeredness cannot be a universal approach that anyone welcomes (7), and it is more important to establish a relationship that is appropriate for the patient by interpreting it as a form of adaptability that responds sensitively to the patient's needs.

The results of the evaluation of each item's importance by the subjects indicate the relative importance of each cluster. Medical students who participated in the study rated “Patient-centered” as the most important. Next came “Partnership”, followed by “Fragile relationship”, “Consumer service”, “Inevitable relationship”, and “Doctor-oriented”. As a result, Korean medical students have balanced concepts of doctor–patient relations that have changed from the past to the present, but they think the most important relationship is the patient-centered relationship, which has been the most emphasized in recent years, as evidenced by the fact that medical students have recognized patient-centered care and patient-centered communication as the most important factors in medical care (17–21). On the other hand, the lowest level of importance was assigned to the category “Doctor-centered.” According to the study, Korean medical students showed a doctor-centered attitude compared to American medical students (19). However, in this study students recognize the past concept of the doctor-centered relationship, but they do not consider it as an important concept in the current situation. In other words, students are aware that the authoritarian attitude stemming from the so-called unilateral communication of doctors, who have the right and obligation to decide on the treatment of patients themselves, should be avoided.

In conclusion, medical students have a complex concept of doctor–patient relationships, from traditional doctor–patient relationships to patient-centered and partnership relationships, which are currently emphasized. This perception would have been taught indicatively or implicitly in medical school. In the future, medical schools will need to provide students with a continuous education to recognize the advantages and disadvantages of various concepts around doctor–patient relationships and to form patient-doctor relationships suitable for specific medical situations. This study is different from previous studies, which only presented dichotomous results in which the patient-centeredness is as high and as low as the score shown through the patient-centeredness measurement tool (20). In order to learn more details about the doctor–patient concept of medical students, more studies using qualitative research methods such as concept mapping, which are directly conducted by the subjects, need to be conducted.

This study has limitations in generalizing the results of the study in that it only targeted 4th year medical students. It was assumed that students who experienced a doctor-patient relationship in a hospital for more than one year would be suitable as subjects of this study in Korean medical education situation. However, in future research, it is necessary to examine the difference in perception between grades by targeting all grades of medical school or to analyze the perceptions of all medical college students in an integrated way.

## Data Availability Statement

The raw data supporting the conclusions of this article will be made available by the authors, without undue reservation.

## Ethics Statement

The studies involving human participants were reviewed and approved by IRB approval No. 1041386-202007-HR-37-02. The patients/participants provided their written informed consent to participate in this study.

## Author Contributions

SK and SY contributed to the analysis and interpretation of the data, read the manuscript critically, and participated in revising the manuscript. SK took the lead in the writing of the manuscript. SY and SK drafted the manuscript. All authors contributed to setting the study design and to collecting the data, approved the final manuscript to be published and agreed to be accountable for all aspects of the work.

## Funding

This work was supported by a 2019 Humanities Social-Science Arts Journal Research Promotion of Pusan National University. Pusan National University staff had no role in the design of the study, nor in the collection, analysis, and interpretation of the data, nor in the preparation of this manuscript.

## Conflict of Interest

The authors declare that the research was conducted in the absence of any commercial or financial relationships that could be construed as a potential conflict of interest.

## Publisher's Note

All claims expressed in this article are solely those of the authors and do not necessarily represent those of their affiliated organizations, or those of the publisher, the editors and the reviewers. Any product that may be evaluated in this article, or claim that may be made by its manufacturer, is not guaranteed or endorsed by the publisher.

## Abbreviations

GSM: Group similarity measure
MDS: Multidimensional scaling
HCA: Hierarchical cluster analysis.
